# Adherence to a Healthy Lifestyle and the Risk of All-Cause Mortality and Cardiovascular Events in Individuals With Diabetes: The ARIC Study

**DOI:** 10.3389/fnut.2021.698608

**Published:** 2021-07-05

**Authors:** Dongze Li, Yu Jia, Jing Yu, Yi Liu, Fanghui Li, Yanmei Liu, Qinqin Wu, Xiaoyang Liao, Zhi Zeng, Zhi Wan, Rui Zeng

**Affiliations:** ^1^Department of Emergency Medicine and National Clinical Research Center for Geriatrics, Research Laboratory of Emergency Medicine, Disaster Medicine Center, West China Hospital, Sichuan University, Chengdu, China; ^2^West China School of Nursing, West China Hospital, Sichuan University, Chengdu, China; ^3^Department of Cardiology, West China Hospital, Sichuan University, Chengdu, China; ^4^Chinese Evidence-Based Medicine Center, West China Hospital, Sichuan University, Chengdu, China; ^5^Health Management Center, West China Hospital, Sichuan University, Chengdu, China; ^6^Department of General Practice and National Clinical Research Center for Geriatrics, West China Hospital, Sichuan University, Chengdu, China

**Keywords:** diabetes, healthy lifestyle, mortality, cardiovascular events and mortality, cohort study

## Abstract

**Objective:** The relationship between combined healthy lifestyle and cardiovascular (CV) events in diabetes is unclear. We aim to investigate the association between a healthy lifestyle score (HLS) and the risk of mortality and CV events in diabetes.

**Methods:** We examined the associations of six lifestyle factors scores (including healthy diet, moderate alcohol and regular coffee intakes, never smoking, physical activity, and normal weight) with diabetes in the Atherosclerosis Risk in Communities (ARIC) study of 3,804 participants with diabetes from the United States at baseline. Primary outcomes included all-cause mortality, CV mortality, and composite CV events (heart failure hospitalizations, myocardial infarction, fatal coronary heart disease, and stroke).

**Results:** Among these diabetic participants, 1,881 (49.4%), 683 (18.0%), and 1,600 (42.0%) cases of all-cause mortality, CV mortality, and CV events were documented, respectively, during the 26 years of follow-up. Further, the prevalence of these adverse events became lower with the increase of HLS (all *P* < 0.001). In the risk-factors adjusted Cox regression model, compared to participants with HLS of 0, participants with HLS of 2 had significant lower risk of all-cause mortality (HR = 0.811, 95% CI: 0.687–0.957, *P* = 0.013), CV mortality (HR = 0.744, 95% CI: 0.576–0.962, *P* = 0.024), and CV events (HR = 0.789, 95% CI: 0.661–0.943, *P* = 0.009). The association of HLS with CV events was stronger for women than men (*P* for interaction <0.05).

**Conclusion:** Adherence to a healthy lifestyle was associated with a lower risk of CV events and mortality in diabetics. Our findings suggest that the promotion of a healthy lifestyle would help reduce the increasing healthcare burden of diabetes.

**Clinical Trial Registration:** https://clinicaltrials.gov, Identifier: NCT00005131.

## Introduction

Diabetes affects more than 400 million people globally (~8.4% of the world's population). This number may rise dramatically to 693 million (9.9%) by 2045 with an aging population and the prevalence of unhealthy lifestyles worldwide (1). The cardiovascular (CV) system is the primary system affected by diabetes, and despite major therapeutic advances that have improved outcomes over the past 2 decades, cardiovascular disease (CVD) remains the leading cause of morbidity and mortality in patients with diabetes (1, 2). Compared to the general population, the risk for CVD in diabetes is two to five times higher, independent of other risk factors (3). The global burden of diabetes is exacerbated by the fact that a large proportion of patients develop CVD, which affects their life expectancy and accounts for the majority of their healthcare costs (4, 5).

Although some studies indicate that pharmacologic management is considerably effective in improving CV events, studies of pharmacologic treatment in secondary prevention show contradictory results in its ability to reduce CV events; furthermore, it is costly and may have unpleasant side effects (6). In contrast, adherence to a healthy lifestyle has become a mainstream approach to lower CV burden through primary and secondary prevention emphasized by guidelines (2, 6, 7). In epidemiological studies, ~72.6% of incident diabetes were attributable to a combination of CV risk factors over a period of about a decade (8). This includes an adherence to multiple healthy lifestyle factors including lower weight, healthy dietary patterns, being physically active, not smoking, and moderate alcohol consumption. All of these have been associated with up to a 90% reduction of incidences of diabetes (9, 10), nearly an 80% reduction of coronary heart disease (CHD), a 67.9% reduction in ischemic heart disease, and a 50% reduction in ischemic strokes (11–13). In addition, non-adherence to healthy lifestyles contributes to 60.7% of all-cause mortality, and 71.7% of for CV mortality in general population (14).

Common pathways associated with a healthy lifestyle may play a protective role in reducing risks of CV events, such as anti-inflammatory action, inhibition of platelet activity, improved insulin resistance, and enhanced antioxidant activity (7, 15, 16). Adherence to a healthy lifestyle, which alleviates comprehensive CV risk factors, is more likely to improve CV events in patients with diabetes. However, previous studies largely focused on the association between adherence to healthy lifestyle and total mortality (17–19), and little is known if cardiac protective effects persist throughout the course of diabetes. Therefore, there is a need for well-controlled studies of the potential effects of lifestyle interventions to better understand how a healthy lifestyle can be implemented in clinical practice for diabetic individuals.

Thus, this study aimed to examine the associations of a combination of modifiable, healthy lifestyle factors with the risks of all-cause mortality and CV events among individuals with type 1 or type 2 diabetes in a large, long-term follow-up cohort study.

## Methods

### Study Population

This study was based on the Atherosclerosis Risk in Communities (ARIC) study. ARIC data are available through The National Heart, Lung, and Blood Institute Biologic Specimen and Data Repository Information Coordinating Center. ARIC is a large prospective study covering four communities (Minneapolis, MN; Forsyth County, North Carolina; Washington County, Maryland; and Jackson, MS) in the United States. At baseline (1987–1989), 15,792 community residents aged between 45 and 64 participated in this study. Follow-up was conducted every 3 years to update medical history, lifestyle, CV events and other health-related events. The institutional review committee at each site approved the study, and participants provided informed consent.

In this study, data from 1987 were used as the baseline for ARIC, including 4,464 participants with type 1 or type 2 diabetes at baseline. Diabetes is defined as a blood glucose concentration ≥ 126 mg/dL, a non-fasting blood glucose concentration ≥ 200 mg/dL, self-reported history of diabetes, or use of diabetes drugs in the past 2 weeks. Thus, type 1 and type 2 diabetes cannot be distinguished according to the definition in ARIC study. We excluded participants without a complete assessment of a healthy lifestyle (*N* = 117), patients with diagnosed with a myocardial infarction, stroke, or malignant tumor (*N* = 264; a diagnosis of these diseases might cause changes in lifestyle habits; in addition, they contributed significantly to mortality), patients with implausible caloric intakes (≤ 500 kcal/day or >5,000 kcal/day, *N* = 66), those who were neither white nor black (*N* = 24), and participants without covariates (*N* = 184). The final participant count was 3,804.

### Definition of the Healthy Lifestyle Score

The healthy lifestyle score (HLS) was calculated based on six factors: healthy diet, moderate alcohol, coffee consumption, physical activity, normal body weight, and being a non-smoker. Each health lifestyle factor added a score of 1 to the HLS, thus HLS ranged from 0 to 6. In ARIC, participants' dietary intake was assessed by the 66 food item-frequency semi-quantitative Food Frequency questionnaire (FFQ) by Willett et al. (20). Participants were asked about the frequency of specified portions of 66 foods, and their daily nutrient intake was calculated by multiplying the nutrient content of a specific part of each food by its daily intake frequency, and then adding up all foods. Dietary quality was assessed using the Mediterranean diet scale of Trichopoulou et al. (21). The original score was based on the intake of 9 items, and possible scores on the Mediterranean diet scores ranged from 0 to 9. This diet is rich in vegetables, fruits, extra virgin olive oil, nuts, legumes and whole grains, while containing low to medium levels of animal products. It has been identified as a high-quality diet for the prevention of diabetes (22). We removed the alcohol score from the Mediterranean Diet since alcohol intake was measured as an independent component of HLS. We defined a healthy diet as the highest two quintiles of Mediterranean diet scores which was scored 1, whereas the lowest three quintiles scored 0, as in previous methods (9, 18, 23, 24). The mean (SD) of Mediterranean diet score for total population was 4.0 (1.8), and mean (SD) was 5.7 (0.9) for the top two quintiles of Mediterranean diet score. Guidelines in the United States define moderate alcohol intakes as consuming 5–15 g of alcohol per day for women and 5–30 g per day for men (25). Moderate alcohol intakes scored 1 point, whereas intakes outside of this range scored 0. Coffee consumption was defined as drinking ≥ 2 servings per day and scored 1 point, while drinking <2 servings per day scored 0. According to previous reports, general and diabetic individuals with a regular intake of ≥ two cups per day has a lower risk of CV and all-cause mortality (26, 27). In Visit 1, 3, and 5, participants answered questions about participation in up to four exercise activities, and how often they participated, by the Baecke Questionnaire (28). A score of 1 point was assigned to those performing healthy physical activity, defined as physical activity of ≥ 150 min per week of vigorous intensity or ≥ 300 min of moderate intensity (≥15 MET-hour/week), while 0 point was assigned for those who did not accomplish enough exercise (29). Trained staff measured the participants' weight and height during follow-up, which was used to calculate body mass index (BMI). Normal body weight was defined as a BMI from 18.5 to 24.9 kg/m^2^ according to the WHO standard and scored 1 point, whereas the range of BMI <18.5 or ≥ 25.0 kg/m^2^ was scored 0 (30). Smoking status was assessed according to smoking history, never smoked was scored 1 point, while past smoking or present smoking was scored 0.

Because lifestyle factors may be affected by the risk of death over a long period of time, in order to obtain an accurate assessment of a healthy lifestyle, detailed and repeated measurements of healthy lifestyle factors were applied to calculate the HLS. The Mediterranean Diet score, alcohol and coffee consumption, and physical activities were calculated by the average of 3-year repeated measurements. To minimize reverse causality, we applied the lifelong maximum BMI.

### Definition of Outcomes

Our primary results contained CV events, CV disease mortality, and all-cause mortality. CV events and CV deaths were determined by annual telephone calls with participants or agents, active monitoring of local discharge and death records, and the National Death Index. CV events were defined as myocardial infarction, fatal coronary heart disease, stroke, or hospitalized heart failure (31). Stroke was defined as the discharge diagnostic code [International Classification of Diseases ninth revision (ICD-9), code 430-438]. The incident stroke was defined as the first known or probable stroke incident among participants with a history of stroke without a doctor's diagnosis at the baseline (32). Heart failure was recorded according to discharge diagnostic code (ICD-9, code 428). CV disease mortality was defined as deaths with an ICD-9 code of 390 to 459. All-cause mortality was defined as death from any cause.

### Covariate Assessment

At baseline, participants' socio-demographic information (age, sex, race, income, and education), health behaviors (smoking, alcohol consumption, physical activity, dietary intake), and medicine use (antihypertensive or diabetic drug use) were collected through a standard self-reporting questionnaire. The concentration of total cholesterol was determined by using an enzymatic method. The trained technicians measured the blood pressure of the subjects three times, then took the average values of the second and third times. We defined hypertension as systolic blood pressure ≥ 140 mmHg, diastolic blood pressure ≥ 90 mmHg, or using antihypertensive drugs in the past 2 weeks. The concentration of blood glucose was determined by improved hexokinase or glucose-6-phosphate dehydrogenase method.

### Statistical Analysis

Categorical variables are reported as frequencies and percentages and were compared using a chi-squared test. Continuous variables were reported as the mean ± standard deviation and compared using an analysis of variance.

The Cox proportional hazards regression model was used to assess the relationship of HLS with the time to the all-cause mortality, CV mortality, and CV events for individuals with diabetes. The group with an HLS of 0 was set as reference group in the Cox regression model. To further determine if these relationships were independent of risk factors, the model was adjusted according to demographic variables (age, sex, race, education) (< high school, high school, or >high school), annual household income (<16,000; 16,000–35,000; >35,000 US$), heart rate, systolic blood pressure, total cholesterol, high density lipoprotein cholesterol, low density lipoprotein cholesterol, triglycerides, creatinine, blood glucose, and total caloric intake. In addition, after adjusting for confounding factors, the Cox regression analysis was performed to evaluate the association between HLS and all-cause mortality in different subgroups of age, sex, and race, as well as their interactions. In order to explain the different results in the subgroup of sex, the correlation of BMI with adjusted hazard ratios (HRs) for all-cause mortality was analyzed using linear splines with five evenly spaced knots in men and women.

A two-tailed *P* < 0.05 was considered significant for all tests. All statistical analyses were performed using SPSS version 26.0 (IBM Corp, Armonk, NY, USA), and R software 3.5.0 (Vienna, Austria).

## Results

### Baseline Characteristics

There were 3,804 participants with diabetes included in our prospective analyses. The diabetics with HLS of 1, 2, 3, 4, 5, and 6 were as follows, respectively: 344 (9.0%), 1,243 (32.7%), 1,289 (33.9%), 682 (17.9%), 210 (5.5%), 33 (0.9%), and 3 (0.1%). As the proportion of diabetics in the population with HLS of 5 or 6 is too low to analyze, the population was divided into five groups with an HLS of 0, 1, 2, 3, and 4–6, respectively. Baseline (1987–1989) characteristics are described and compared in Table 1. Participants with a higher HLS were more likely to be male and Caucasian, have lower levels of biomarkers for CV disease, have more healthy behaviors such as physical activity and a healthy diet (i.e., more vegetables, fruits, nuts, and whole grains, less beverages, and higher Mediterranean diet score); moderate drinking and coffee consumption, keeping fit, and never smoking (*P* < 0.001 for all).

**Table 1:** Baseline (1987–1989) participant characteristics grouped by healthy lifestyle score.

**Characteristic**	**Healthy lifestyle score**					***P***
	**0**	**1**	**2**	**3**	**4–6**	
*N*	344 (9.0)	1,243 (32.7)	1,289 (33.9)	682 (17.9)	246 (6.5)	
**Demographic variables**							
Age, years	53.6 ± 5.4	53.9 ± 5.7	54 ± 5.7	54.3 ± 5.8	54.1 ± 5.7	0.462
Male sex	153 (44.5)	514 (41.4)	582 (45.2)	331 (48.5)	129 (52.4)	<0.001
Race (Black)	184 (53.5)	570 (45.9)	410 (31.8)	112 (16.4)	22 (8.9)	0.003
MET-hour, /week	2.3 ± 4.2	5.6 ± 11.2	12.4 ± 16.8	20 ± 17.7	26.7 ± 16.3	<0.001
Education						<0.001
Less than high school	148 (43)	414 (33.3)	346 (26.8)	122 (17.9)	33 (13.4)	
High school	94 (27.3)	369 (29.7)	408 (31.7)	213 (31.2)	89 (36.2)	
College	102 (29.7)	460 (37)	535 (41.5)	347 (50.9)	124 (50.4)	
Smoking						<0.001
Never	0 (0)	457 (36.8)	644 (50)	411 (60.3)	163 (66.3)	
Ever	210 (61)	480 (38.6)	358 (27.8)	168 (24.6)	42 (17.1)	
Current	134 (39)	306 (24.6)	287 (22.3)	103 (15.1)	41 (16.7)	
Drinking						<0.001
Never	88 (25.6)	407 (32.7)	416 (32.3)	166 (24.3)	52 (21.1)	
Ever	111 (32.3)	335 (27)	264 (20.5)	95 (13.9)	19 (7.7)	
Current	145 (42.2)	501 (40.3)	609 (47.2)	421 (61.7)	175 (71.1)	
Income, US$						<0.001
<16,000	153 (44.5)	443 (35.6)	350 (27.2)	120 (17.6)	32 (13)	
16,000–35,000	101 (29.4)	405 (32.6)	430 (33.4)	234 (34.3)	77 (31.3)	
>35 000	90 (26.2)	395 (31.8)	509 (39.5)	328 (48.1)	137 (55.7)	
**Physiological and Lab variables**							
Body mass index, kg/m^2^	32 ± 5.1	31.1 ± 5.3	30.3 ± 6	28.4 ± 5.1	26.2 ± 4.7	<0.001
Total cholesterol, mmol/l	5.6 ± 1.1	5.6 ± 1.2	5.6 ± 1.1	5.6 ± 1.1	5.5 ± 1.1	0.849
HDL, mmol/l	1.2 ± 0.4	1.2 ± 0.4	1.2 ± 0.4	1.3 ± 0.4	1.3 ± 0.4	<0.001
LDL, mmol/l	3.6 ± 1	3.6 ± 1	3.6 ± 1	3.6 ± 1	3.5 ± 1	0.689
Triglycerides, mg/dl	1.8 ± 1.2	1.9 ± 1.5	1.8 ± 1.2	1.7 ± 1.1	1.7 ± 1.4	0.001
Creatinine, mg/dl	1.2 ± 0.5	1.2 ± 0.8	1.1 ± 0.4	1.1 ± 0.2	1.1 ± 0.2	0.099
Blood glucose, mmol/l	7.9 ± 3.9	7.8 ± 3.7	7.6 ± 3.7	7.1 ± 3	7 ± 3.2	<0.001
**Dietary intake**							
Total calories, kcal	1,563 ± 623	1,550 ± 590	1,644 ± 618	1,692 ± 594	1,814 ± 634	<0.001
Coffee intake, servings/d	0.4 ± 0.5	1.1 ± 1.7	1.7 ± 2.2	2.5 ± 2.2	3.1 ± 2.2	<0.001
Alcohol intake, g/d	6.1 ± 18.9	4.2 ± 11.8	4.9 ± 12.7	5.5 ± 9.2	7.6 ± 8.9	<0.001
Fruits, servings/d	1.2 ± 1.3	1.4 ± 1.4	1.6 ± 1.4	1.7 ± 1.3	1.8 ± 1.1	<0.001
Vegetables, servings/d	1.5 ± 1.1	1.6 ± 1.1	1.8 ± 1.2	2 ± 1.3	2.3 ± 1.6	<0.001
Red and processed meat, servings/d	1.2 ± 0.8	1.2 ± 0.8	1.2 ± 0.8	1.2 ± 0.9	1.2 ± 0.9	0.329
Nuts, servings/d	0.6 ± 0.5	0.6 ± 0.5	0.7 ± 0.6	0.8 ± 0.6	0.8 ± 0.6	<0.001
Sweetened beverages, servings/d	1.5 ± 1.4	1.2 ± 1.1	1.0 ± 1.1	0.9 ± 0.9	0.8 ± 0.9	<0.001
Whole grains, g/d	0.5 ± 0.6	0.5 ± 0.5	0.6 ± 0.6	0.6 ± 0.5	0.8 ± 0.9	<0.001
Mediterranean diet score	3.0 ± 1.5	3.6 ± 1.7	4.1 ± 1.7	4.5 ± 1.7	5.1 ± 1.6	<0.001

*Values are expressed as N (%), mean ± SD. SBP, systolic blood pressure; DBP, diastolic blood pressure; HDL, high density lipoprotein; LDL, low density lipoprotein*.

### Healthy Lifestyle Pattern and Adverse Outcome

**Figure 1:**
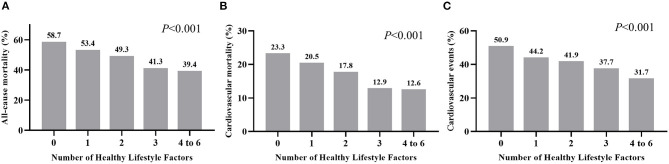
Incidence of all-cause mortality **(A)**, cardiovascular mortality **(B)**, and cardiovascular events **(C)** grouped by number of healthy factors for diabetic individuals.

During the 26 years of follow-up, we documented 1,881 (49.4%), 683 (18.0%), and 1,600 (42.0%) cases of all-cause mortality, CV mortality, and CV events among diabetic participants. Participants with a higher HLS had a lower incidence of these adverse outcomes (*P* < 0.001, Figure 1). In risk-factors adjusted in the Cox regression model (Table 2), compared to participants without any healthy lifestyle factors, participants with only 2 healthy lifestyle factors had a significantly lower risk of all-cause mortality (HR = 0.811, 95% CI: 0.687–0.957, *P* = 0.013), CV mortality (HR = 0.744, 95% CI: 0.576–0.962, *P* = 0.024), and CV events (HR = 0.789, 95% CI: 0.661–0.943, *P* = 0.009); while participants with an HLS ≥ 4 demonstrated a significant lower HR of all-cause mortality (0.593, 95% CI: 0.389–0.905, *P* = 0.015), CV mortality (0.593, 95% CI: 0.389–0.905, *P* = 0.015), and CV events (0.677, 95% CI: 0.511–0.896, *P* = 0.006) than an HLS of 0.

**Table 2:** Adjusted hazard ratios for the association of healthy lifestyle score with all-cause mortality, cardiovascular mortality, and cardiovascular events.

**Healthy lifestyle score**	**Unadjusted**		**Model 1**		**Model 2**	
	**HR (95% CI)**	***P***	**HR (95% CI)**	***P***	**HR (95% CI)**	***P***
All-cause mortality		<0.001		<0.001		<0.001
0	Ref.	–	Ref.	–	Ref.	–
1	0.873 (0.746–1.022)	0.090	0.874 (0.747–1.024)	0.095	0.939 (0.798–1.106)	0.451
2	0.760 (0.648–0.890)	0.001	0.787 (0.671–0.923)	0.003	0.811 (0.687–0.957)	0.013
3	0.580 (0.484–0.694)	<0.001	0.615 (0.512–0.740)	<0.001	0.719 (0.593–0.871)	0.001
4–6	0.545 (0.428–0.695)	<0.001	0.597 (0.466–0.764)	<0.001	0.593 (0.389–0.905)	0.015
Cardiovascular mortality		<0.001		<0.001		<0.001
0	Ref.	–	Ref.	–	Ref.	–
1	0.851 (0.662–1.094)	0.209	0.857 (0.667–1.102)	0.230	0.862 (0.670–1.109)	0.249
2	0.696 (0.540–0.898)	0.005	0.756 (0.585–0.977)	0.032	0.744 (0.576–0.962)	0.024
3	0.464 (0.343–0.628)	<0.001	0.543 (0.398–0.739)	<0.001	0.514 (0.377–0.701)	<0.001
4–6	0.447 (0.295–0.677)	<0.001	0.560 (0.367–0.856)	0.007	0.593 (0.389–0.905)	0.015
Cardiovascular events		<0.001		<0.001		0.008
0	Ref.	–	Ref.	–	Ref.	–
1	0.776 (0.654–0.920)	0.003	0.789 (0.666–0.936)	0.007	0.849 (0.712–1.013)	0.069
2	0.700 (0.590–0.830)	<0.001	0.741 (0.624–0.880)	0.001	0.789 (0.661–0.943)	0.009
3	0.539 (0.445–0.654)	<0.001	0.599 (0.492–0.729)	<0.001	0.708 (0.577–0.869)	0.001
4–6	0.441 (0.338–0.576)	<0.001	0.499 (0.380–0.655)	<0.001	0.677 (0.511–0.896)	0.006

*Model 1: adjusted by age, sex, race*.

*Model 2: adjusted by model 1 plus education (< high school, high school, or >high school), annual household income (<16,000, 16,000–35,000, >35,000 US$), heart rate, systolic blood pressure, total calorie intake, total cholesterol, high density lipoprotein cholesterol, low density lipoprotein cholesterol, triglycerides, creatinine, and blood glucose*.

*Unexplained variables are regarded as continuous variables. HR, hazard ratio; CI, confidence interval*.

### Subgroup Analysis

**Figure 2:**
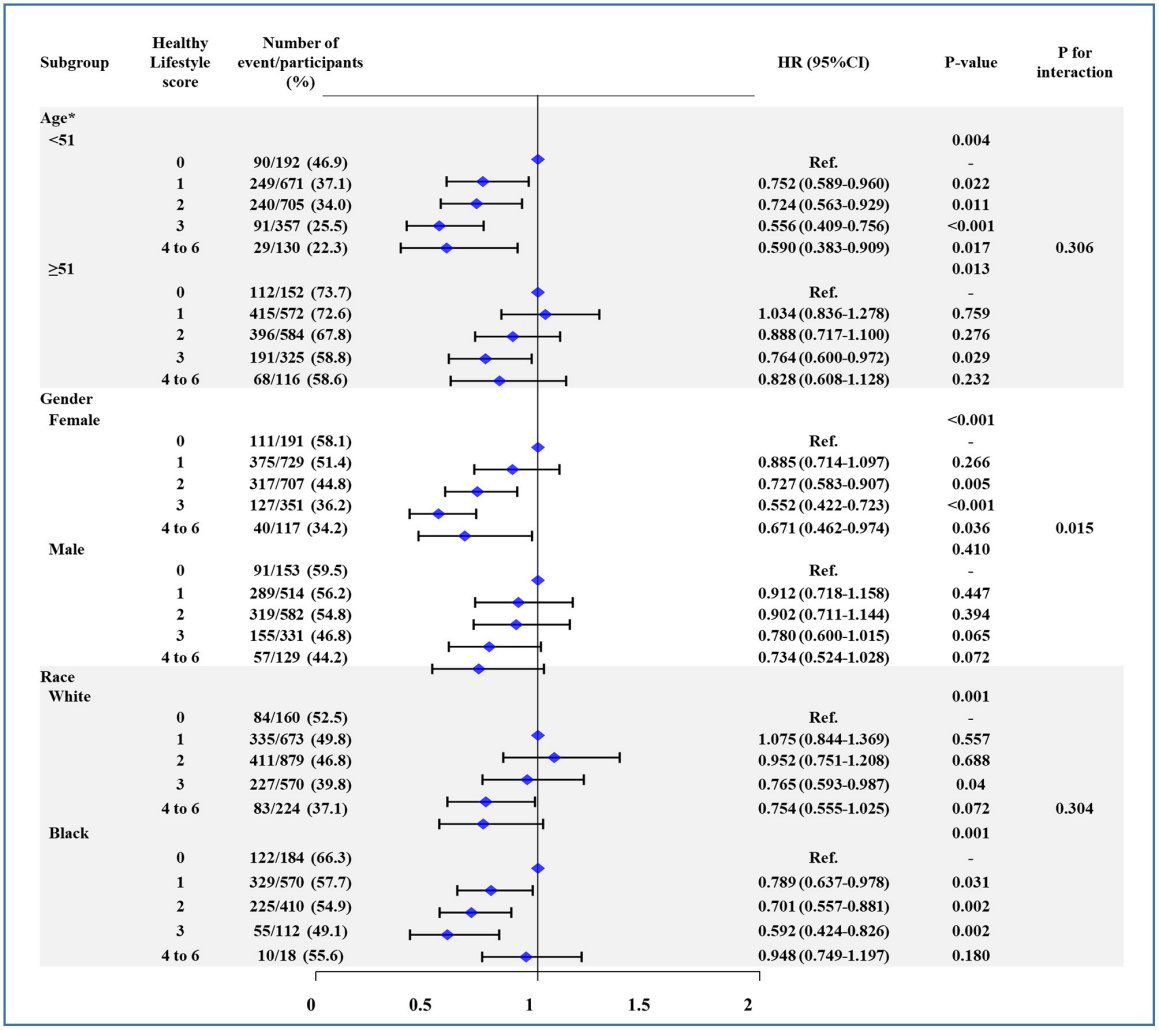
The association between healthy lifestyle score (HLS) and all-cause mortality in subgroup of age, sex, and race. Age was divided by median. Models were adjusted by age, sex, center-race, education (< high school, high school, or >high school), annual household income (<16,000; 16,000–35,000; >35,000 US$), heart rate, systolic blood pressure, total caloric intake, total cholesterol, high density lipoprotein cholesterol, low density lipoprotein cholesterol, triglycerides, creatinine, and blood glucose. CI, confidence interval. HR, hazard ratio.

Figure 2 shows subgroup analyses stratified by age, sex, and race. As is evident, there was no interaction for age and race, with similar risk of all-cause mortality (Figure 2), CV mortality (Supplementary Figure 1), and CV events (Supplementary Figure 2) between younger (<51 years) vs. older adults (≥51 years) and white vs. black individuals. However, the risk of all three outcomes was affected by sex (*P* < 0.05 for interaction), where the association between higher HLS and risk reduction was significant for women.

### Factors Analysis Between Men and Women

**Figure 3:**
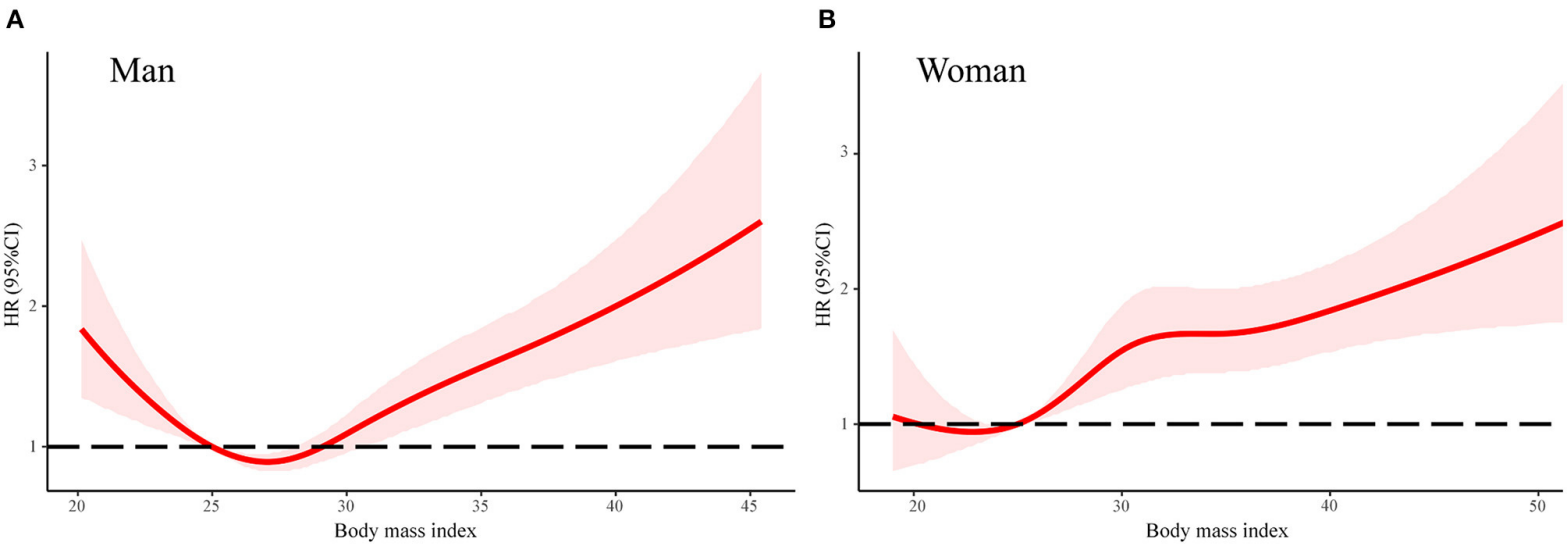
The association between body mass index and all-cause mortality in linear splines curve for men **(A)** and women **(B)**. Models were adjusted by age, sex, center-race, education (< high school, high school, or >high school), annual household income (<16,000; 16,000–35,000; >35,000 US$), heart rate, systolic blood pressure, total caloric intake, total cholesterol, high density lipoprotein cholesterol, low density lipoprotein cholesterol, triglycerides, creatinine, and blood glucose. CI, confidence interval. HR, hazard ratio.

In order to explore the differences between men and women and the association between HLS and adverse events, we analyzed the factors of six binary variables of HLS. As Table 3 shows, in risk-factors adjusted Cox regression model, six binary variables of HLS were all significantly associated with all-cause mortality for women. Meanwhile, only a healthy diet, physical activity, and never smoking had a significantly protective effect against all-cause mortality (Table 3). Moreover, the associations of normal body weight with all-cause mortality were stronger for women than men (*P* = 0.002 for interaction). Given the unexpected result that men with normal weight (WHO standard: BMI is 18.5–25 kg/m^2^) did not have a lower risk of all-cause mortality, we conducted a linear spline between the association of BMI and the adjusted HR for all-cause mortality (Figure 3). There was a U-shape and a linear association between BMI and HR of all-cause mortality in men and women, respectively. For men, participants with the lowest risk of all-cause death were overweight (WHO standard: BMI is 25–30 kg/m^2^).

**Table 3:** Multivariable Cox regression model for association of healthy lifestyle factors and all-cause mortality for man and woman.

**Lifestyle factors**	**Men**		**Women**		***P* for interaction**
	**HR (95% CI)**	***P***	**HR (95% CI)**	***P***	
**MDS**					0.846
Lowest 3 quintiles	Ref.	-	Ref.	-	
Highest 2 quintiles	0.721 (0.556–0.934)	0.013	0.775 (0.661–0.91)	0.002	
**Alcohol intake**					0.451
Not moderate	Ref.	–	Ref.	–	
Moderate	0.924 (0.846–1.008)	0.074	0.904 (0.821–0.996)	0.041	
**Body mass index**					0.002
<18.5 or ≥ 25 kg/m^2^	Ref.	–	Ref.	–	
18.5–24.9 kg/m^2^	0.956 (0.794–1.150)	0.634	0.644 (0.534–0.777)	<0.001	
**Physical activity**					0.946
<15 MET-hour/week	Ref.	–	Ref.	–	
≥15 MET-hour/week	0.795 (0.695–0.908)	0.001	0.730 (0.623–0.854)	<0.001	
**Consuming coffee**					0.037
<2 servings/day	Ref.	–	Ref.	–	
≥ 2 servings/day	0.898 (0.787–1.025)	0.112	0.795 (0.690–0.916)	0.001	
**Smoking**					0.783
Former or current	Ref.	–	Ref.	–	
Never	0.653 (0.560–0.762)	<0.001	0.780 (0.687–0.884)	<0.001	

*Moderate alcohol intake was defined as consuming 5–15 grams of alcohol per day for women and 5–30 grams per day for men. Multivariable Cox regression models were adjusted by age, sex, race, education (< high school, high school, or >high school), annual household income (<16,000, 16,000–35,000, >35 000 US$), heart rate, systolic blood pressure, total calorie intake, total cholesterol, high density lipoprotein cholesterol, low density lipoprotein cholesterol, triglycerides, creatinine, and blood glucose. HR, hazard ratio; CI, confidence interval; MDS, Mediterranean diet score*.

## Discussion

In this community-based cohort study, a healthy lifestyle—including a healthy diet (reflected by the highest two quintiles of the Mediterranean diet scores), never smoking, moderate alcohol intake (5–15 g for men; 5–30 g for women), regular coffee consumption (≥2 cups/d), physical activity (≥15 MET-hour/week), and normal body weight (BMI: 18.5–24.9 kg/m^2^)—was associated with a lower risk of all-cause mortality, CV mortality, and CV events among diabetic participants. This association also suggests that participants with a higher HLS had a gradually decreased (81 to 59%) risk of adverse outcomes. Therefore, assuming causal relations, the risk of these three adverse outcomes might be reduced by one fifth to two fifths when all diabetic participants have an HLS ≥ 2.

In the subgroup analysis, we found that an association between HLS and adverse events of diabetic patients were consistent with age and race (Caucasian vs. African Americans), but this association was stronger in women than in men. Analyzing the difference in the distribution of single lifestyle factors might explain this phenomenon. As shown, in risk-factors adjusted in the Cox regression model, all six healthy lifestyle factors are significantly associated with all-cause mortality in women, while only healthy diet, physical activity, and never smoking had significant protective effects against mortality in men. Moreover, it appears as though men cannot benefit by having a normal body weight. According to our analysis of linear splines, there was a non-linear (U-shape) association between BMI and risk of all-cause mortality in men. Like several studies have shown, the lowest risk group for men with diabetes mellitus was the overweight group (33–35). In fact, there is growing evidence that overweight patients with CVD survive longer than their normal weight counterparts, an effect called the “obesity paradox” (36). Inconsistently, some studies did not support the obesity paradox for diabetic individuals (37, 38). Moreover, according to previous studies, women with a low-risk lifestyle had a lower risk of cancer, CV disease, diabetes, and mortality than men; however, the reasons for this disparity are not totally clear (23, 39–41).

It is well-established that a healthy lifestyle related to a lower risk of CVD and mortality in most healthy people (24, 42). Regarding associations between lifestyle and health outcomes among diabetic patients, previous studies largely focused on total mortality (17–19). In the current study, we addressed a major limitation in previous studies by the average of 3-year repeated measurements of dietary and lifestyle factors to capture potential changes of lifestyle practices. Moreover, we evaluated the associations of an overall 6-factor healthy lifestyle (different from previous 4 or 5-factor healthy lifestyle and usually did not include coffee consuming) and CV events (including myocardial infarction, fatal coronary heart disease, stroke, or hospitalized heart failure) and CV mortality, in addition to all-cause mortality. Our findings were in line with an observational study which included 867 newly diagnosed diabetic patients, in which a greater number of healthy behavior changes within the 1st year of diagnosis were associated with a lower risk of CV outcomes (43).

Substantial evidence supports the benefits of lifestyle modification for the prevention of all-cause mortality and CV events. Moderate alcohol consumption, never or quitting smoking, physical activity, regular consumption of coffee, staying fit, and following a Mediterranean Diet have been reported to reduce the risk of diabetes and improve CV health in patients with diabetes (22, 26, 44–46). All of these healthy lifestyle factors were related to insulin resistance (47–52), which is a key pathophysiological mechanism for increasing the morbidity and mortality of diabetes. Further, the modified lifestyle factors have been shown to effectively alter the lipid profile and, thus, could alleviate pathways related to CV disease (53–56).

Since the 1980's—especially since 2013—the American Heart Association, the American College of Cardiology, and the European Society of Cardiology have launched clinical practice guidelines on lifestyle management to prevent CV disease and diabetes, as well as improving CV health (57). This study provides additional evidence that adherence to a comprehensive healthy lifestyle could alleviate the risk of CVD and mortality for people with diabetes. Thus, HLS may serve as a useful and simple tool to screen for high risk of CV events and mortality in primary prevention. Through risk stratification, diabetic individual lifestyle intervention and risk management could be further adopted by healthcare professionals.

### Limitations

There are some limitations which should be mentioned. First, although this study came from the ARIC with a large sample size that has been regularly updated for over 30 years, these results should be further confirmed by other prospective cohorts and randomized controlled studies. Second, only 1% of diabetic participants had an HLS of 5 to 6 (this is significantly lower than the general population in other studies) (11, 23); thus, analysis for these populations was limited. Third, the detailed and repeated measurements of lifestyle factors in this study enabled us to take dynamic changes over time into account, but it was also limited by its reliance on self-reported lifestyle factors. Therefore, measurement errors are inevitable. Fourth, some potential confounding factors, such as medication, health insurance, diabetic complications, and quality of treatment, may not have been adequately adjusted. Fifth, top two quintiles of healthy diet score were usually defined as healthy diet for general or diabetic population in previous studies (9, 18, 23, 24), but there is no evidence that any diet score greater than a certain point can be classified as healthy diet for diabetic individuals. Finally, the cardiac metabolic risk of type 1 diabetes is different from that of type 2 diabetes, but they are indistinguishable according to the definition of diabetes in ARIC study.

## Conclusion

In this cohort, adherence to an overall healthy lifestyle was associated with a lower risk of CV events and mortality in diabetic individuals. Given the increasing prevalence of diabetes and continuous high risk of adverse outcomes, our findings suggest that the promotion of a healthy lifestyle would help reduce the gradually increasing healthcare burdens via non-drug interventions. Future research should measure a more accurate range of healthy lifestyle factors for diabetic individuals, such as alcohol consumption and BMI.

## Data Availability Statement

ARIC data are available through the NIH NHLBI-sponsored Biologic Specimen and Data Repository Information Coordinating Center (BioLINCC) at https://biolincc.nhlbi.nih.gov.

## Ethics Statement

The studies involving human participants were reviewed and approved by The institutional review committee at each site approved the study. Written informed consent to participate in this study was provided by the participants' legal guardian/next of kin.

## Author Contributions

DL, YJ, and RZ designed the research. DL, YJ, QW, and YML analyzed the data. DL and YJ wrote the first draft of the manuscript. JY, YL, FL, XL, ZZ, and ZW reviewed the manuscript and provided critical scientific input. RZ holds the primary responsibility for the final content of the manuscript. All the authors have approved the final draft of the manuscript.

## Conflict of Interest

The authors declare that the research was conducted in the absence of any commercial or financial relationships that could be construed as a potential conflict of interest.

## Supplementary Material

Supplementary Figure 1 The association between healthy lifestyle score (HLS) and cardiovascular mortality in subgroup of age, sex, and race. The detailed description is the same as Figure 1.

Supplementary Figure 2 The association between healthy lifestyle score (HLS) and cardiovascular events in subgroup of age, sex, and race. The detailed description is the same as Figure 2.

## Abbreviations

ARIC: Atherosclerosis Risk in Communities
BMI: body mass index
CVD: cardiovascular disease
FFQ: Food Frequency questionnaire
HLS: healthy lifestyle score.
