# Supplementary material for: Adherence to a Healthy Lifestyle and the Risk of All-Cause Mortality and Cardiovascular Events in Individuals With Diabetes: The ARIC Study
Source: Front Nutr. 2021 Jul 5;8:698608. doi: 10.3389/fnut.2021.698608 (PMC8287067; doi:10.3389/fnut.2021.698608)
Supplement: Supplementary Figure 1 — The association between healthy lifestyle score (HLS) and cardiovascular mortality in subgroup of age, sex, and race. The detailed description is the same as Figure 1. [file Data_Sheet_1.PDF]

Subgroup	Healthy Lifestyle score	Number of event/participants (%)	HR (95%CI)	P-value	P for interaction
**Age***					
<51				0.139	
	0	33/192 (17.2)	Ref.	-	
	1	87/671 (13.0)	0.782 (0.522-1.170)	0.231	
	2	83/705 (11.8)	0.791 (0.524-1.192)	0.262	
	3	26/357 (7.3)	0.509 (0.298-0.869)	0.013	
	4 to 6	8/130 (6.2)	0.557 (0.253-1.228)	0.147	0.306
≥51				0.003	
	0	47/152 (30.9)	Ref.	-	
	1	168/572 (29.4)	0.926 (0.670-1.282)	0.645	
	2	146/584 (25.0)	0.745 (0.535-1.037)	0.081	
	3	62/325 (19.1)	0.542 (0.368-0.798)	0.002	
	4 to 6	23/116 (19.8)	0.629 (0.379-1.044)	0.073	
**Gender**					
Female				<0.001	
	0	50/191 (26.2)	Ref.	-	
	1	147/729 (20.2)	0.741 (0.537-1.023)	0.068	
	2	111/707 (15.7)	0.545 (0.389-0.764)	<0.001	
	3	35/351 (10.0)	0.305 (0.195-0.475)	<0.001	
	4 to 6	13/117 (11.1)	0.463 (0.248-0.864)	0.016	0.005
Male				0.62	
	0	30/153 (19.6)	Ref.	-	
	1	108/514 (21.0)	1.048 (0.698-1.572)	0.822	
	2	118/582 (20.3)	1.078 (0.720-1.616)	0.715	
	3	53/331 (16.0)	0.872 (0.553-1.376)	0.556	
	4 to 6	18/129 (14.0)	0.800 (0.442-1.448)	0.46	
**Race**					
White				0.001	
	0	27/160 (16.9)	Ref.	-	
	1	115/673 (17.1)	1.147 (0.754-1.746)	0.522	
	2	132/879 (15.0)	0.930 (0.614-1.409)	0.734	
	3	67/570 (11.8)	0.632 (0.403-0.992)	0.046	
	4 to 6	23/224 (10.3)	0.570 (0.327-0.995)	0.048	0.232
Black				0.003	
	0	57/184 (31.0)	Ref.	-	
	1	140/570 (24.6)	0.738 (0.537-1.015)	0.061	
	2	97/410 (23.7)	0.661 (0.472-0.926)	0.016	
	3	21/112 (18.8)	0.451 (0.270-0.752)	0.002	
	4 to 6	4/18 (22.2)	0.820 (0.609-1.036)	0.15	

0 0.5 1 1.5 2

Supplementary Figure 1

Subgroup	Healthy Lifestyle score	Number of event/participants (%)	HR (95%CI)	P-value	P for interaction
Age*					
<51				0.065	
	0	88/192 (45.8)	Ref.	-	
	1	251/671 (37.4)	0.773 (0.517-1.156)	0.209	
	2	251/705 (35.6)	0.758 (0.504-1.141)	0.184	
	3	104/357 (29.1)	0.490 (0.289-0.831)	0.008	
	4 to 6	27/130 (20.8)	0.454 (0.206-0.998)	0.050	0.088
≥51				0.011	
	0	87/152 (57.2)	Ref.	-	
	1	299/572 (52.3)	0.929 (0.672-1.285)	0.658	
	2	289/584 (49.5)	0.773 (0.555-1.075)	0.126	
	3	153/325 (47.1)	0.579 (0.394-0.851)	0.005	
	4 to 6	51/116 (44.0)	0.618 (0.372-1.026)	0.063	
Gender					
Female				<0.001	
	0	96/191 (50.3)	Ref.	-	
	1	315/729 (43.2)	0.771 (0.557-1.067)	0.117	
	2	271/707 (38.3)	0.570 (0.405-0.802)	0.001	
	3	124/351 (35.3)	0.350 (0.221-0.552)	<0.001	
	4 to 6	30/117 (25.6)	0.586 (0.312-1.099)	0.096	0.007
Male				<0.001	
	0	79/153 (51.6)	Ref.	-	
	1	235/514 (45.7)	1.091 (0.722-1.647)	0.679	
	2	269/582 (46.2)	1.127 (0.748-1.700)	0.567	
	3	133/331 (40.2)	0.773 (0.582-1.027)	0.075	
	4 to 6	48/129 (37.2)	0.704 (0.489-1.015)	0.060	
Race					
White				<0.001	
	0	70/160 (43.8)	Ref.	-	
	1	281/673 (41.8)	1.074 (0.706-1.634)	0.738	
	2	352/879 (40.0)	0.875 (0.578-1.324)	0.526	
	3	207/570 (36.3)	0.610 (0.390-0.955)	0.031	
	4 to 6	67/224 (29.9)	0.509 (0.292-0.889)	0.018	
Black				0.001	0.499
	0	109/184 (59.2)	Ref.	-	
	1	269/570 (47.2)	0.747 (0.541-1.033)	0.078	
	2	188/410 (45.9)	0.664 (0.470-0.938)	0.020	
	3	50/112 (44.6)	0.407 (0.237-0.700)	0.001	
	4 to 6	7/18 (38.9)	0.762 (0.642-0.906)	0.002	

0 0.5 1 1.5 2

Supplementary Figure 2
